# Non-cancer disease prevalence and association with occupational radiation exposure among Korean radiation workers

**DOI:** 10.1038/s41598-021-01875-2

**Published:** 2021-11-17

**Authors:** Soojin Park, Dal Nim Lee, Young Woo Jin, Eun Shil Cha, Won-Il Jang, Sunhoo Park, Songwon Seo

**Affiliations:** 1 National Radiation Emergency Medical Center, Korea Institute of Radiological and Medical Sciences, 75 Nowon-ro, Nowon-gu, Seoul, 01812 South Korea; 2 Department of Preventive Medicine, Korea University College of Medicine, Seoul, South Korea; 3 Department of Radiation Oncology, Korea Institute of Radiological and Medical Sciences, Seoul, South Korea; 4 Department of Pathology, Korea Institute of Radiological and Medical Sciences, Seoul, South Korea

**Keywords:** Diseases, Health occupations

## Abstract

Radiation-induced cancer risks have known stochastic effects; however, regarding non-cancer diseases, evidence of risk at low radiation doses remains unclear. We aimed to identify underlying characteristics concerning non-cancer disease prevalence and determine associations with radiation dose among Korean radiation workers. Using a nationwide baseline survey, 20,608 workers were enrolled. Data concerning participant demographics, occupational characteristics, lifestyle, and lifetime prevalence of non-cancer diseases were linked to a national dose registry. We compared non-cancer disease prevalences in the Korean general population with those in this cohort and undertook a dose–response analysis concerning the cumulative dose. Hyperlipidemia (10.6%), circulatory (9.6%), and respiratory (4.1%) system diseases, followed by thyroid diseases (3.5%), had the highest prevalences, with hyperlipidemia, thyroid diseases, and hepatitis prevalence being higher in the cohort than in the general population. Radiation doses were associated with elevated prevalences of most diseases; however, associations were attenuated and not significant after adjusting for confounders, except for musculoskeletal system diseases (prevalence odds ratio [POR]/10 mSv, 1.03; 95% confidence interval [CI] 1.00–1.07) and cataracts (POR/10 mSv, 1.04; 95% CI 1.00–1.07). Further studies are warranted to investigate the causality of those non-cancer diseases involving more varied confounders such as physical and psychosocial stresses and ultraviolet light.

## Introduction

Radiation effects can generally be categorized into stochastic effects and tissue reactions (i.e., deterministic effects): radiation-induced cancer risk is the best known stochastic effect^1^, and non-cancer diseases are often considered as tissue reactions with a threshold causing temporary or permanent changes in the human body^2^. However, issues concerning risk in non-cancer diseases have emerged as a long-term consequence of ionizing radiation.

The Hiroshima and Nagasaki Atomic Bomb Survivors Life-Span Study (LSS study) and some radiation workers’ studies have suggested potential stochastic effects for non-cancer diseases^3–5^. In the LSS study, a higher risk of death was observed in all non-cancer diseases combined and in several sub-diseases (i.e., circulatory, respiratory, and digestive diseases) with increasing levels of radiation exposure^5–7^. These findings have also been supported by some radiation worker studies. The recent International Nuclear Workers Study (INWORKS), which included three countries (France, the United Kingdom, and the United States), also reported further evidence that occupational radiation exposure may increase the risk of non-cancer diseases, particularly circulatory diseases^4^. Previously, it has been established that radiation exposure to the lens, known to be one of the most radiation-sensitive tissues in the body, can lead to cataracts^8,9^. The International Commission on Radiological Protection (ICRP) classified cataract development as a non-probabilistic effect, and the threshold was set to 0.5 Gy^10^. However, recent radiation worker studies have reported a positive dose–response association for cataracts, suggesting possibilities of stochastic effects^11,12^. A study among Chernobyl clean-up workers also identified significant dose–response relationships in different types of cataracts^13^. Moreover, for studies concerning medical radiation exposure in interventional cardiologists, the risk of posterior subcapsular cataract (PSC) development was approximately three times higher than that in control groups not exposed to radiation. Furthermore, a United States study involving radiologists also identified an increased cataract risk in line with radiation dose^12,14,15^. However, consistency among the findings from studies of non-cancer diseases appears to be much lower than that of cancer, and relevant evidence concerning radiation-induced non-cancer diseases has not yet been accumulated due to limited epidemiological findings.

The Korean Radiation Workers Study (KRWS) was launched in 2016 to assess the health effects of occupational exposure among radiation workers in various nuclear-related occupations^16,17^. In a previously published paper, we presented baseline characteristics on cancer risk from the KRWS^16^ and, by extension, this study aimed to evaluate the association between occupational radiation exposure and the prevalence of non-cancer diseases among Korean radiation workers.

## Methods

### Study population

Data used in this study were derived from the KRWS, and the study design and population are described in detail elsewhere^16,17^. In summary, we conducted nationwide surveys on 42,607 active radiation workers via mandatory radiation safety education programs between 2016 and 2017, and among them, 35,789 workers responded to the surveys. After excluding 15,181 subjects who responded more than once or who would not be able to complete follow-up due to unidentified personal identification numbers or disagreement with study participation, a total of 20,608 workers were enrolled in the cohort, accounting for approximately 50% of the target population of this study. The cohort consisted of workers from facility-based occupations including nuclear power plants (30.7%, n = 6328), industry (18.9%, n = 3886), industrial radiography (17.1%, n = 3517), education and research institutes (15.3%, n = 3149), medical institutes (14.0%, n = 2887), public institutes (3.3%, n = 676), and military (0.8%, n = 165). We also collected data concerning participant demographics, occupational characteristics, lifestyle, and lifetime prevalence of non-cancer diseases (diseases of the circulatory system, diseases of the respiratory system, diseases of the musculoskeletal system, thyroid diseases, hyperlipidemia, cataracts, diabetes mellitus, and hepatitis) from a self-reported questionnaire of the survey that we linked to a national dose registry of radiation workers.

### Occupational exposure to radiation

Occupational radiation doses for workers in this study have been described in detail in a previously published study^16^. In brief, all radiation workers were issued a personal dosimeter to measure their personal radiation dose equivalent (Hp^10^), and these measurements were reported quarterly to the Central Registry for Radiation Workers Information (CRRWI) throughout each year. We obtained data from the CRRWI concerning external radiation doses of workers in 1984 (when the CRRWI was initiated) through to the first quarter of 2017.

### Definition of the lifetime prevalence of non-cancer diseases

Information from the baseline survey was used to measure self-reported non-cancer diseases in radiation workers who were classified as having diseases if they answered “Yes” to the question “Have you ever been diagnosed with any of the following diseases by a physician at a hospital?” The non-cancer diseases in the survey included diseases of the circulatory system (hypertension, stroke, myocardial infarction, and angina), diseases of the respiratory system (pulmonary tuberculosis, asthma, and chronic bronchitis), diseases of the musculoskeletal system (rheumatoid arthritis and osteoporosis), thyroid diseases, hyperlipidemia, cataracts, diabetes mellitus, and hepatitis.

### Statistical analysis

To compare the lifetime prevalence of non-cancer diseases between radiation workers and the general population in Korea, age- (5-year intervals) and sex-specific standardized prevalence ratios (SPRs) and 95% confidence intervals (CIs) were used. Information concerning the lifetime prevalence of non-cancer diseases in the Korean general population was obtained from the Korea National Health and Nutrition Examination Survey (KNHANES) in 2016, a nationally representative study of the Korean population, which is a population-based, cross-sectional epidemiological survey that has been performed since 1998 and is designed to assess the health-related behavior, health condition, and nutritional status of Koreans (https://knhanes.kdca.go.kr/knhanes/eng/)^18^. The average response rate of the KNHANES in 2016 was 75.4%, and the health interview questionnaires, which were used in this study, were collected via self-administration^19,20^. Since the questionnaire about the lifetime prevalence of non-cancer diseases in radiation workers was identical to those of the KNHANES with an overlap of their survey periods, the lifetime prevalences of non-cancer diseases from those surveys were comparable.

For internal comparisons, prevalence odds ratios (PORs) and 95% CIs were estimated according to six categories of cumulative doses (a priori defined as 0, 0.10–0.99, 1.00–4.99, 5.00–19.99, 20.00–49.99, and ≥ 50.0 mSv) with a trend test and PORs per unit dose (10 mSv) using logistic regression. The PORs were adjusted for sex, age, occupation, duration of employment, smoking status, alcohol consumption, regular exercise, body mass index (BMI), and night-shift work. Statistical analysis was conducted using SAS version 9.4 (SAS Institute, Cary, NC) software.

All study participants provided written informed consent prior to study enrollment. This study received ethical approval from the Institutional Review Board of the Korea Institute of Radiological and Medical Sciences (IRB No. K-1603-002-034); all methods were performed in accordance with the relevant guideline and regulations.

## Results

The mean cumulative dose in the cohort was 11.8 (standard deviation [SD] 28.8) mSv, and the median was 0.6 mSv during the employment period from 1984 to 2017. Workers with a cumulative dose of 0 mSv comprised 38% of the total, followed by 0.10–0.99 (15%), 1.00–4.99 (15%), 5.00–19.99 (15%), 20.00–49.99 (9%), and ≥ 50.0 (7%) mSv.

Among 20,608 Korean radiation workers, the prevalence of hyperlipidemia was highest overall in terms of the number of patients diagnosed (10.6%), followed by circulatory system (9.6%), respiratory system (4.1%), and thyroid (3.5%) diseases. The prevalence of most non-cancer diseases was higher in men than in women, but the prevalence of respiratory and thyroid diseases was higher in women (Table 1).

**Table 1:** Prevalence of non-cancer diseases in Korean radiation workers (n = 20,608).

Diseases	Total (n = 20,608)		Men (n = 17,831)		Women (n = 2777)	
	n	PR (%)	n	PR (%)	n	PR (%)
**Diseases of the circulatory system**	1855	(9.6%)	1801	(10.8%)	54	(2.1%)
Hypertension	1759	(9.3%)	1709	(10.4%)	50	(1.9%)
Stroke	37	(0.2%)	31	(0.2%)	6	(0.2%)
Myocardial infarction	69	(0.4%)	64	(0.4%)	5	(0.2%)
Angina	103	(0.5%)	96	(0.6%)	7	(0.3%)
**Diseases of the respiratory system**	768	(4.1%)	654	(4.0%)	114	(4.4%)
Pulmonary tuberculosis	386	(2.0%)	351	(2.2%)	35	(1.3%)
Asthma	287	(1.5%)	223	(1.4%)	64	(2.5%)
Chronic bronchitis	202	(1.1%)	162	(1.0%)	40	(1.5%)
**Diseases of the musculoskeletal system**	184	(1.0%)	150	(0.9%)	34	(1.3%)
Rheumatoid arthritis	114	(0.6%)	98	(0.6%)	16	(0.6%)
Osteoporosis	92	(0.5%)	68	(0.4%)	24	(0.9%)
Thyroid diseases^a^	669	(3.5%)	501	(3.1%)	168	(6.5%)
Hyperlipidemia	2005	(10.6%)	1919	(11.7%)	86	(3.3%)
Cataracts	156	(0.8%)	141	(0.9%)	15	(0.6%)
Diabetes mellitus	563	(3.0%)	543	(3.3%)	20	(0.8%)
Hepatitis	322	(1.7%)	306	(1.9%)	16	(0.6%)

*PR* prevalence rate.

^a^Including benign thyroid tumor, thyroid nodule, thyroid goiter, thyroiditis, hyperthyroidism, and hypothyroidism.

The prevalence of non-cancer diseases according to demographic and occupational characteristics is summarized in Supplementary Table S1. For all diseases, prevalence increased with age, with the prevalence of diseases of the circulatory system, hyperlipidemia, and diabetes mellitus increasing markedly in overweight and obese workers. When comparing workers based on their occupations, nuclear power plant workers had a higher prevalence of non-cancer diseases than workers of other occupations. The prevalence of non-cancer diseases was also highest in workers who had performed radiation work over a longer period and in workers who had performed night-shift work for > 10 years (Supplementary Table S1). The SPRs and 95% CIs comparing non-cancer disease prevalence in radiation workers with that in the general population are summarized in Table 2. In men, the SPRs of diseases of the circulatory, respiratory, and musculoskeletal systems, cataracts, and diabetes mellitus were significantly decreased (SPRs, 0.42–0.95, p < 0.05), whereas the SPRs of hyperlipidemia, thyroid diseases, and hepatitis were significantly increased (SPRs, 1.20–2.31, p < 0.05) in our cohort. In women, the SPRs of all diseases tended to decrease except for that of thyroid diseases (SPR, 1.66; 95% CI 1.30–1.42).

**Table 2:** Age- and sex-specific standardized prevalence ratios (SPRs).

Diseases	Men (n = 17,831)		Women (n = 2777)		Total (n = 20,608)	
	SPR	(95% CI)	SPR	(95% CI)	SPR	(95% CI)
Diseases of the circulatory system^a^	0.95	(0.90–0.99)	0.88	(0.68–1.11)	0.94	(0.90–0.99)
Diseases of the respiratory system^b^	0.80	(0.74–0.87)	0.91	(0.74–1.11)	0.81	(0.75–0.88)
Diseases of the musculoskeletal system^c^	0.73	(0.62–0.85)	0.51	(0.36–0.69)	0.67	(0.58–0.77)
Thyroid diseases^d^	2.31	(2.11–2.52)	1.66	(1.42–1.93)	2.10	(1.95–2.27)
Hyperlipidemia	1.36	(1.30–1.42)	0.97	(0.78–1.20)	1.34	(1.28–1.40)
Cataracts	0.42	(0.36–0.50)	0.79	(0.44–1.30)	0.44	(0.38–0.52)
Diabetes mellitus	0.84	(0.77–0.92)	0.66	(0.40–1.02)	0.83	(0.77–0.91)
Hepatitis	1.20	(1.07–1.34)	0.80	(0.46–1.29)	1.17	(1.04–1.30)

*CI* confidence interval.

^a^Including hypertension, stroke, myocardial infarction, and angina.

^b^Including pulmonary tuberculosis and asthma.

^c^Including rheumatoid arthritis and osteoporosis.

^d^Including benign thyroid tumor, thyroid nodule, thyroid goiter, thyroiditis, hyperthyroidism, and hypothyroidism.

Results of the dose–response analysis among the radiation workers are shown in Supplementary Table S2 and Table 3. In the univariate analysis to assess the relationship between radiation dose and the prevalence of each non-cancer disease, an increased prevalence of most diseases was associated with an increased cumulative dose. However, the associations were not statistically significant after adjusting for confounders such as sex, age, occupation, duration of employment, smoking status, alcohol consumption, regular exercise, and night-shift work, except for diseases of the musculoskeletal system (POR per 10 mSv, 1.03; 95% CI 1.00–1.07) and cataracts (POR per 10 mSv, 1.04; 95% CI 1.00–1.07). On the other hand, a decreased prevalence of thyroid diseases was associated with an increased cumulative dose (POR per 10 mSv, 0.96; 95% CI 0.93–0.99). In the sub-analysis stratified according to occupation and birth year, the prevalence rates of diseases of the musculoskeletal system in nuclear power plant workers (POR per 10 mSv, 1.05; 95% CI 1.01–1.10) and of cataracts in workers born before 1960 (POR per 10 mSv, 1.06; 95% CI 1.01–1.11) were positively associated with an increasing cumulative dose even after adjusting for confounders (Table 3).

**Table 3:** Prevalence odds ratios (PORs) per 10 mSv according to occupation and birth year.

	Adjusted POR^a^ per 10 mSv (95% CI)							
	Diseases of the circulatory system^b^	Diseases of the respiratory system^c^	Diseases of the musculoskeletal system^d^	Thyroid diseases^e^	Hyperlipidemia	Cataracts	Diabetes mellitus	Hepatitis
**Occupation**								
Public institute	1.05 (0.84–1.31)	0.81 (0.38–1.73)	0.64 (0.19–2.16)	1.15 (0.82–1.61)	1.00 (0.79–1.27)	0.00 (0–113.65)	1.27 (0.97–1.66)	0.85 (0.48–1.53)
Education and research institute	0.95 (0.73–1.23)	0.93 (0.62–1.40)	1.05 (0.63–1.74)	0.66 (0.38–1.15)	0.99 (0.76–1.28)	0.85 (0.36–2.00)	0.99 (0.68–1.43)	1.05 (0.62–1.80)
Industrial radiography	1.00 (0.96–1.05)	0.99 (0.91–1.07)	0.99 (0.83–1.19)	0.88 (0.74–1.05)	1.00 (0.95–1.04)	0.91 (0.72–1.14)	1.00 (0.93–1.09)	0.93 (0.81–1.06)
Industry	1.00 (0.84–1.17)	1.01 (0.80–1.29)	0.98 (0.52–1.83)	0.96 (0.64–1.44)	1.05 (0.91–1.22)	1.33 (0.94–1.86)	0.91 (0.67–1.24)	1.12 (0.80–1.56)
Nuclear power plant	1.00 (0.97–1.02)	1.01 (0.97–1.04)	1.05 (1.01–1.10)	0.97 (0.93–1.00)	1.01 (0.99–1.03)	1.03 (0.99–1.08)	1.01 (0.98–1.04)	0.99 (0.95–1.03)
Medical institute	0.92 (0.84–1.00)	0.99 (0.88–1.11)	1.09 (0.90–1.31)	1.09 (0.98–1.21)	0.95 (0.88–1.03)	0.98 (0.72–1.32)	0.99 (0.87–1.14)	0.95 (0.76–1.17)
**Birth year**								
1960 or earlier	0.98 (0.95–1.01)	1.00 (0.94–1.06)	1.04 (0.98–1.11)	1.01 (0.97–1.06)	0.99 (0.96–1.02)	1.06 (1.01–1.11)	1.00 (0.97–1.04)	1.00 (0.94–1.06)
1961–1970	0.99 (0.96–1.02)	1.01 (0.96–1.06)	1.07 (1.00–1.13)	0.90 (0.84–0.96)	1.00 (0.98–1.03)	0.94 (0.85–1.04)	1.02 (0.99–1.06)	0.95 (0.88–1.04)
1971–1980	1.03 (0.99–1.07)	1.03 (0.97–1.09)	1.04 (0.94–1.15)	0.99 (0.92–1.06)	1.02 (0.99–1.06)	1.11 (0.98–1.27)	1.00 (0.93–1.07)	1.02 (0.94–1.11)
1981 or later	1.03 (0.94–1.12)	0.82 (0.70–0.96)	0.74 (0.42–1.29)	0.97 (0.82–1.15)	1.01 (0.92–1.10)	0.89 (0.59–1.34)	0.90 (0.70–1.16)	0.98 (0.83–1.15)

*CI* confidence interval.

^a^Prevalence odds ratios were adjusted for sex, age, occupation, duration of employment, smoking status, alcohol status, regular exercise, body mass index, and night-shift work.

^b^Including hypertension, stroke, myocardial infarction, and angina.

^c^Including pulmonary tuberculosis and asthma.

^d^Including rheumatoid arthritis and osteoporosis.

^e^Including benign thyroid tumor, thyroid nodule, thyroid goiter, thyroiditis, hyperthyroidism, and hypothyroidism.

## Discussion

In this study, we investigated the association between occupational radiation exposure and the prevalence of non-cancer diseases among Korean radiation workers. We observed a significantly lower prevalence of most non-cancer diseases in radiation workers compared with the general population, and our results are similar to those of other radiation worker studies. A study of workers engaged in the uranium fuel cycle in France reported that the standardized mortality ratio (SMR) of these workers was lower than that of the general population with respect to circulatory, respiratory, and musculoskeletal diseases^21^. In a pooled cohort study of United States nuclear workers, a significantly lower mortality rate was observed in relation to circulatory and respiratory diseases than in the general population^22,23^. Moreover, the SMRs of most non-cancer diseases in men were significantly lower in a study of medical diagnostic radiation workers in Korea^24^. These results are often explained as the health worker effect.

However, we observed that the SPRs for thyroid diseases, hyperlipidemia, and hepatitis were increased. The increased SPRs for thyroid diseases can possibly be attributed to thyroid screening, with similar increased thyroid cancer incidence rates observed among radiation workers in several studies^16,25–28^. The increased SPR for hyperlipidemia may have been due to improved access to healthcare services for workers through the workers’ general health examinations (WGHEs) system, where an examination for hyperlipidemia is included in the second health examination and constitutes a mandatory component of workers’ regular health checkups. The increased SPR for hepatitis among the radiation workers may be attributed to a larger range of hepatitis investigations compared with that in the general population. The survey question concerning hepatitis for the radiation workers did not specify the hepatitis type (i.e., any type of hepatitis could have been reported); however, the survey question for the general population included only hepatitis B and C. Hepatitis A, which was not included in the survey for the general population, has been continuously reported, with 3–9 per 100,000 persons reported as having hepatitis A between 2014 and 2018, and the average estimated prevalence of antibodies for hepatitis A in Korean adults (range, 20–89 years) in 2018 was approximately 65%^29,30^. Therefore, although hepatitis B comprises most of the hepatitis cases in South Korea, inclusion of the prevalence rates concerning hepatitis A may have led to a higher prevalence of hepatitis in radiation workers compared with that in the general population.

In a dose–response analysis, a negative association was observed in thyroid diseases after adjusting for confounding factors. Given that our study population was in active service and mostly in good health at the time of the survey, this finding may imply a health worker effect. Although we included the employment period of individual workers as a confounder to minimize a possible health worker effect, it may not have been completely adjusted.

We found a modestly significant increase in the dose–response association in relation to cataracts in workers born before 1960. Although the mean cumulative dose of workers with cataracts in our cohort was 27.0 (SD 59.5) mSv (range, 0–392.9 mSv; median, 1.93 mSv), which is below the current threshold of 0.5 Gy for tissue reactions^10^, we cannot exclude radiation-induced cataracts in terms of stochastic effects, given that we are uncertain whether radiation-induced cataracts can be solely considered as tissue reactions^31^. Particularly, PSCs are known to be possibly due to radiation exposure rather than aging^32–34^. In the Occupational Cataract Lens Opacities in Interventional Cardiology (O’CLOC study) study involving French interventional cardiologists, the odds ratios (ORs) of PSC in the exposed groups were increased, even after adjusting for confounding factors (OR 3.85, 95% CI 1.30–11.40), compared with those in the control group. Moreover, the ORs increased in line with an earlier start year of interventional cardiology activity and a longer working duration^35^. In the International Atomic Energy Agency (IAEA) cataract study, greater changes were reported in the subcapsular lenses of cardiologists exposed to occupational radiation compared with the control groups (relative risk 2.6, 95% CI 1.2–5.4)^36^.

Non-cancer diseases of the musculoskeletal system were associated with radiation doses, particularly among nuclear power plant workers in this cohort. Diseases of the musculoskeletal system are suggested as late effects of radiation, with incidences occurring months to years after exposure to radiation^10^; however, epidemiological evidence and the biological mechanisms involved remain unclear. The musculoskeletal diseases may be related to various risk factors. The main causes of musculoskeletal diseases involve lifting heavy items, working in awkward postures, and performing similar tasks repetitively^37^. These ergonomic risk factors have been reported to be the most important factors contributing to the incidence of musculoskeletal diseases; however, psychosocial factors have also been emphasized recently^38–40^. Rheumatoid arthritis (RA), a subgroup of musculoskeletal system diseases and the most common autoimmune disease^41^, has been associated with a family history of RA and psychological stresses such as depression and anxiety^42,43^. In the nuclear industry, particularly in nuclear power plants that include multiple power generation, cooling, and containment systems, more sophisticated operations and maintenance are required^44^. These physical and psychosocial stresses may be more likely to increase the burden on the musculoskeletal system. Similarly, a previous study on musculoskeletal disorders among radiation workers also suggested the disease incidence was due to occupational characteristics or individual factors rather than an association with radiation exposure^45^.

Our study had some limitations. First, the prevalence was measured using a self-reporting method. Self-reported data have been shown to be less reliable than other sources of information, such as medical records, and can lead to information or recall bias^46^. However, it should be noted that registry-based prevalence rates for diabetes mellitus and hypertension—which are among the most prevalent diseases in Korea—are 3% and 10%, respectively, among the total employer-provided policyholders in Korea^47^. These rates are similar to the self-reported prevalence rates in our cohort, which shows that our self-reported disease prevalence is unlikely to be highly biased. Moreover, the younger age of the participants in our cohort (mean age, 38.3 years) may have been an advantage in leading to less recall bias in the self-reported questionnaire. Nevertheless, linking disease registries such as national insurance claims data is required in any future studies to clarify data reliability. Second, since prevalence and confounding factors were investigated based on a cross-sectional survey, a temporal correlation could not be identified. Specifically, because we could not identify the time of disease onset, we cannot exclude the possibility of disease onset prior to employment. However, the average age at which radiation work began in this cohort was less than 30 years, which is not high enough for the onset of chronic diseases. Considering that the prevalences of hypertension and hyperlipidemia, which were the non-cancer diseases with the highest prevalences in this cohort, are 1.1% and 1.5%, respectively, among Korean adults in their 20s according to the KNHANES in 2016 (data available online at https://knhanes.kdca.go.kr/knhanes/eng/), most non-cancer diseases in this cohort were likely to have occurred during employment. Although this study aimed to investigate the association between baseline characteristics and prevalence rates concerning non-cancer diseases in relation to radiation doses for radiation workers, further studies are needed to investigate radiation-induced non-cancer diseases based on a cohort study design that includes disease incidence and organ-absorbed doses, including internal doses. Third, exposure misclassification cannot be excluded because we could not identify radiation doses prior to 1984, indicating that radiation doses of participants who were employed before 1984 were possibly underestimated; based on the survey responses, 290 workers (1.4%) were assumed to have worked before 1984 in this study. Thus, the dose–response association in this study might be overestimated, assuming that increased radiation doses are associated with disease prevalence. Fourth, we cannot rule out selection bias owing to non-participation in this survey. However, considering that selection bias is a common and potentially serious problem particularly in a case–control study^48,49^ and nonresponses in voluntary sampling do not seem to necessarily bias the associations among survey items^50^, nonresponses are not likely to have substantially influenced the exposure-disease associations in this study. In addition, the distributions of the number of workers and radiation doses by occupation types in the cohort did not substantially deviate from those of the target population, and the study population was in active service with a mostly healthy status^16^; therefore, the selection of study subjects would not be highly related to exposure and disease status. Finally, we collected participant demographic and lifestyle data in relation to non-cancer diseases concerning various risk factors; however, we were not able to include other potential risk factors in this study. In particular, cataracts and musculoskeletal diseases, which showed marginally significant associations with radiation doses in our study, may be associated with ultraviolet light, underlying diseases, psychological stress, and sensitivity to autoimmune diseases. Moreover, these associations were weak and still subject to potential bias from residual confounding. Therefore, more detailed data are needed in relation to other confounding factors in future studies.

Despite these limitations, a strength of this study was that various non-cancer diseases were included, such as circulatory diseases and cataracts, and we investigated their associations with radiation doses collected from the national dose registry. Furthermore, data were collected concerning major confounding factors such as cigarette smoking, alcohol consumption, BMI, and night-shift work, which have rarely been adjusted in combination in previous studies involving radiation workers. Therefore, this comprehensive individual information will likely shed more light on the risks of radiation-induced disease among radiation workers.

## Conclusion

This study was the first to investigate the characteristics of non-cancer diseases in terms of radiation doses among Korean radiation workers with nuclear-based occupations. The baseline findings indicated that hyperlipidemia and diseases of the circulatory system were the most prevalent non-cancer diseases in the cohort; however, there was no association between hyperlipidemia or circulatory system diseases and occupational exposure. Diseases of the musculoskeletal system and cataracts were marginally associated with occupational exposure, and this finding warrants further studies to investigate causality involving more varied confounding factors and further information concerning the time of disease onset. This study is likely to enhance understanding in terms of the risk of non-cancer diseases among radiation workers who are typically exposed to low-dose radiation.

## Supplementary Information

Supplementary Tables.

## Data Availability

The raw data of the KRWS are currently not publicly available due to the requirement of participants’ agreement for sharing data to a third party; however, data not containing personal information may be available from the corresponding author on reasonable request. The data of the KNHANES in this article are publicly available on the study website (https://knhanes.kdca.go.kr/knhanes/eng/).
