# Supplementary material for: Non-cancer disease prevalence and association with occupational radiation exposure among Korean radiation workers
Source: Sci Rep. 2021 Nov 17;11:22415. doi: 10.1038/s41598-021-01875-2 (PMC8599676; doi:10.1038/s41598-021-01875-2)
Supplement: Supplementary file 1 — Supplementary Tables. [file 41598_2021_1875_MOESM1_ESM.pdf]

## Non-cancer disease prevalence and association with occupational radiation exposure among Korean radiation workers

Soojin Park · Dal Nim Lee · Young Woo Jin · Eun Shil Cha · Won-Il Jang · Sunhoo Park · Songwon Seo

**Supplementary Table S1** Prevalence of non-cancer diseases according to demographic and occupational characteristics

| Characteristics | Diseases of the circulatory system[a] | | Diseases of the respiratory system[b] | | Diseases of the musculoskeletal system[c] | | Thyroid diseases[d] | | Hyperlipidemia | | Cataracts | | Diabetes mellitus | | Hepatitis | |
|---|---|---|---|---|---|---|---|---|---|---|---|---|---|---|---|---|
| | n | PR (%) | n | PR (%) | n | PR (%) | n | PR (%) | n | PR (%) | n | PR (%) | n | PR (%) | n | PR (%) |
| **Total** | 1855 | (9.6%) | 768 | (4.1%) | 184 | (1.0%) | 669 | (3.5%) | 2005 | (10.6%) | 156 | (0.8%) | 563 | (3.0%) | 322 | (1.7%) |
| **Demographic characteristics** | | | | | | | | | | | | | | | | |
| Sex | | | | | | | | | | | | | | | | |
| Men | 1801 | (10.8%) | 654 | (4.0%) | 150 | (0.9%) | 501 | (3.1%) | 1919 | (11.7%) | 141 | (0.9%) | 543 | (3.3%) | 306 | (1.9%) |
| Women | 54 | (2.1%) | 114 | (4.4%) | 34 | (1.3%) | 168 | (6.5%) | 86 | (3.3%) | 15 | (0.6%) | 20 | (0.8%) | 16 | (0.6%) |
| Age, years | | | | | | | | | | | | | | | | |
| <30 | 114 | (2.3%) | 169 | (3.4%) | 17 | (0.4%) | 78 | (1.6%) | 68 | (1.4%) | 7 | (0.1%) | 10 | (0.2%) | 25 | (0.5%) |
| 30–39 | 419 | (5.9%) | 247 | (3.5%) | 35 | (0.5%) | 193 | (2.8%) | 518 | (7.4%) | 24 | (0.3%) | 96 | (1.4%) | 94 | (1.4%) |
| 40–49 | 621 | (13.7%) | 184 | (4.2%) | 53 | (1.2%) | 202 | (4.6%) | 771 | (17.6%) | 31 | (0.7%) | 192 | (4.4%) | 99 | (2.3%) |
| 50–59 | 530 | (22.6%) | 130 | (5.9%) | 67 | (3.1%) | 163 | (7.5%) | 528 | (24.0%) | 65 | (3.0%) | 209 | (9.4%) | 86 | (3.9%) |
| ≥60 | 171 | (35.2%) | 38 | (8.5%) | 12 | (2.7%) | 33 | (7.6%) | 120 | (27.2%) | 29 | (6.6%) | 56 | (12.5%) | 18 | (4.1%) |
| BMI, kg/m$^2$ | | | | | | | | | | | | | | | | |
| Underweight (<18.5) | 6 | (1.3%) | 19 | (4.1%) | 9 | (2.0%) | 18 | (4.0%) | 11 | (2.4%) | 1 | (0.2%) | 2 | (0.4%) | 0 | (0.0%) |
| Normal weight (18.5–24.9) | 728 | (6.6%) | 463 | (4.3%) | 119 | (1.1%) | 387 | (3.6%) | 898 | (8.4%) | 95 | (0.9%) | 260 | (2.4%) | 165 | (1.5%) |
| Overweight (25.9–29.9) | 811 | (14.3%) | 200 | (3.6%) | 33 | (0.6%) | 198 | (3.6%) | 832 | (15.0%) | 45 | (0.8%) | 207 | (3.8%) | 122 | (2.2%) |
| Obese (≥30) | 188 | (22.1%) | 35 | (4.3%) | 5 | (0.6%) | 20 | (2.5%) | 135 | (16.4%) | 5 | (0.6%) | 47 | (5.7%) | 16 | (2.0%) |
| Education level | | | | | | | | | | | | | | | | |
| Lower than high school graduation | 33 | (25.8%) | 5 | (4.3%) | 4 | (3.5%) | 2 | (1.7%) | 20 | (16.8%) | 3 | (2.6%) | 14 | (11.8%) | 3 | (2.6%) |
| High school graduation | 450 | (10.8%) | 142 | (3.5%) | 43 | (1.1%) | 94 | (2.3%) | 458 | (11.3%) | 38 | (0.9%) | 181 | (4.4%) | 63 | (1.6%) |

| Characteristics | Diseases of the circulatory system[a] | | Diseases of the respiratory system[b] | | Diseases of the musculoskeletal system[c] | | Thyroid diseases[d] | | Hyperlipidemia | | Cataracts | | Diabetes mellitus | | Hepatitis | |
|---|---|---|---|---|---|---|---|---|---|---|---|---|---|---|---|---|
| | n | PR (%) | n | PR (%) | n | PR (%) | n | PR (%) | n | PR (%) | n | PR (%) | n | PR (%) | n | PR (%) |
| College graduation and higher | 1343 | (9.2%) | 604 | (4.2%) | 133 | (0.9%) | 562 | (3.9%) | 1502 | (10.5%) | 113 | (0.8%) | 357 | (2.5%) | 255 | (1.8%) |
| Marital status | | | | | | | | | | | | | | | | |
| Unmarried | 339 | (4.5%) | 263 | (3.5%) | 36 | (0.5%) | 132 | (1.8%) | 276 | (3.7%) | 19 | (0.3%) | 68 | (0.9%) | 67 | (0.9%) |
| Married/living together | 1447 | (12.9%) | 479 | (4.4%) | 141 | (1.3%) | 522 | (4.8%) | 1662 | (15.3%) | 129 | (1.2%) | 463 | (4.3%) | 242 | (2.2%) |
| Other (divorced, widowed, separated) | 39 | (18.3%) | 8 | (4.0%) | 2 | (1.0%) | 8 | (4.0%) | 36 | (17.7%) | 4 | (2.0%) | 14 | (6.8%) | 11 | (5.5%) |
| Regular exercise | | | | | | | | | | | | | | | | |
| No | 721 | (8.6%) | 350 | (4.2%) | 82 | (1.0%) | 282 | (3.4%) | 786 | (9.5%) | 52 | (0.6%) | 209 | (2.5%) | 126 | (1.5%) |
| Yes | 1087 | (10.2%) | 402 | (3.9%) | 98 | (0.9%) | 379 | (3.7%) | 1182 | (11.4%) | 100 | (1.0%) | 341 | (3.3%) | 189 | (1.8%) |
| Smoking status | | | | | | | | | | | | | | | | |
| Never (non-smoker) | 488 | (6.1%) | 319 | (4.0%) | 68 | (0.9%) | 314 | (4.0%) | 510 | (6.5%) | 43 | (0.5%) | 120 | (1.5%) | 102 | (1.3%) |
| Ex-smoker | 518 | (15.4%) | 143 | (4.4%) | 42 | (1.3%) | 152 | (4.7%) | 552 | (16.9%) | 51 | (1.6%) | 155 | (4.8%) | 77 | (2.4%) |
| Yes (current smoker) | 832 | (10.6%) | 303 | (3.9%) | 73 | (1.0%) | 197 | (2.6%) | 931 | (12.2%) | 61 | (0.8%) | 284 | (3.7%) | 143 | (1.9%) |
| Alcohol consumption | | | | | | | | | | | | | | | | |
| No | 257 | (8.5%) | 117 | (4.0%) | 50 | (1.7%) | 135 | (4.6%) | 312 | (10.7%) | 33 | (1.1%) | 101 | (3.4%) | 69 | (2.3%) |
| Yes | 1584 | (9.7%) | 644 | (4.0%) | 133 | (0.8%) | 527 | (3.3%) | 1679 | (10.6%) | 120 | (0.8%) | 454 | (2.9%) | 250 | (1.6%) |
| **Occupational characteristics** | | | | | | | | | | | | | | | | |
| Occupation | | | | | | | | | | | | | | | | |
| Public institute | 65 | (10.3%) | 18 | (3.0%) | 7 | (1.2%) | 24 | (4.0%) | 65 | (10.7%) | 6 | (1.0%) | 27 | (4.5%) | 10 | (1.7%) |
| Education and research institute | 239 | (8.0%) | 147 | (5.0%) | 29 | (1.0%) | 113 | (3.9%) | 224 | (7.6%) | 21 | (0.7%) | 62 | (2.1%) | 40 | (1.4%) |
| Military | 8 | (5.1%) | 6 | (3.9%) | 1 | (0.7%) | 1 | (0.7%) | 8 | (5.1%) | 0 | (0.0%) | 0 | (0.0%) | 0 | (0.0%) |
| Industrial radiography | 309 | (9.2%) | 129 | (3.9%) | 17 | (0.5%) | 41 | (1.3%) | 338 | (10.4%) | 15 | (0.5%) | 103 | (3.1%) | 61 | (1.9%) |
| Industry | 358 | (9.9%) | 119 | (3.4%) | 32 | (0.9%) | 85 | (2.4%) | 461 | (13.1%) | 26 | (0.7%) | 100 | (2.9%) | 45 | (1.3%) |
| Nuclear power plant | 655 | (11.1%) | 241 | (4.2%) | 68 | (1.2%) | 279 | (4.9%) | 691 | (12.0%) | 63 | (1.1%) | 211 | (3.7%) | 132 | (2.3%) |

| Characteristics | Diseases of the circulatory system[a] | | Diseases of the respiratory system[b] | | Diseases of the musculoskeletal system[c] | | Thyroid diseases[d] | | Hyperlipidemia | | Cataracts | | Diabetes mellitus | | Hepatitis | |
|---|---|---|---|---|---|---|---|---|---|---|---|---|---|---|---|---|
| | n | PR (%) | n | PR (%) | n | PR (%) | n | PR (%) | n | PR (%) | n | PR (%) | n | PR (%) | n | PR (%) |
| Medical institute | 221 | (8.1%) | 108 | (4.0%) | 30 | (1.1%) | 126 | (4.7%) | 218 | (8.2%) | 25 | (0.9%) | 60 | (2.2%) | 34 | (1.3%) |
| Calendar year of hiring | | | | | | | | | | | | | | | | |
| 1989 or earlier | 311 | (26.7%) | 80 | (7.3%) | 32 | (3.0%) | 101 | (9.5%) | 302 | (27.9%) | 46 | (4.2%) | 130 | (11.8%) | 44 | (4.0%) |
| 1990–1999 | 465 | (18.6%) | 113 | (4.7%) | 41 | (1.7%) | 146 | (6.1%) | 521 | (21.6%) | 30 | (1.3%) | 148 | (6.2%) | 67 | (2.8%) |
| 2000–2009 | 432 | (10.1%) | 171 | (4.1%) | 40 | (1.0%) | 142 | (3.4%) | 533 | (12.7%) | 29 | (0.7%) | 124 | (3.0%) | 88 | (2.1%) |
| 2010 or later | 647 | (5.7%) | 404 | (3.6%) | 71 | (0.6%) | 280 | (2.5%) | 649 | (5.8%) | 51 | (0.5%) | 161 | (1.4%) | 123 | (1.1%) |
| Age at the start of radiation work | | | | | | | | | | | | | | | | |
| <20 | 56 | (7.6%) | 30 | (4.1%) | 8 | (1.1%) | 16 | (2.2%) | 51 | (7.0%) | 2 | (0.3%) | 17 | (2.3%) | 4 | (0.6%) |
| 20–29 | 889 | (7.7%) | 458 | (4.0%) | 82 | (0.7%) | 365 | (3.2%) | 991 | (8.8%) | 77 | (0.7%) | 251 | (2.2%) | 169 | (1.5%) |
| 30–39 | 555 | (10.8%) | 191 | (3.8%) | 54 | (1.1%) | 201 | (4.0%) | 643 | (12.8%) | 36 | (0.7%) | 175 | (3.5%) | 95 | (1.9%) |
| 40–49 | 215 | (15.1%) | 49 | (3.6%) | 21 | (1.6%) | 56 | (4.2%) | 218 | (16.1%) | 22 | (1.6%) | 64 | (4.7%) | 37 | (2.8%) |
| ≥50 | 140 | (24.9%) | 40 | (7.7%) | 19 | (3.7%) | 31 | (6.1%) | 102 | (19.6%) | 19 | (3.7%) | 56 | (10.6%) | 17 | (3.3%) |
| Employment status | | | | | | | | | | | | | | | | |
| Regular employment | 1561 | (10.1%) | 607 | (4.0%) | 158 | (1.1%) | 584 | (3.9%) | 1790 | (11.8%) | 134 | (0.9%) | 452 | (3.0%) | 271 | (1.8%) |
| Irregular employment (temporary, daily contract) | 279 | (8.4%) | 138 | (4.3%) | 23 | (0.7%) | 75 | (2.3%) | 200 | (6.2%) | 20 | (0.6%) | 105 | (3.2%) | 46 | (1.4%) |
| Duration of employment, years | | | | | | | | | | | | | | | | |
| ≤5 | 635 | (6.0%) | 374 | (3.6%) | 68 | (0.7%) | 266 | (2.6%) | 631 | (6.1%) | 49 | (0.5%) | 170 | (1.6%) | 113 | (1.1%) |
| 5–9 | 312 | (8.5%) | 142 | (3.9%) | 26 | (0.7%) | 119 | (3.3%) | 363 | (10.1%) | 23 | (0.6%) | 85 | (2.4%) | 71 | (2.0%) |
| 10–14 | 226 | (12.1%) | 93 | (5.1%) | 25 | (1.4%) | 70 | (3.9%) | 266 | (14.5%) | 18 | (1.0%) | 57 | (3.1%) | 42 | (2.3%) |
| ≥15 | 682 | (21.1%) | 159 | (5.2%) | 65 | (2.1%) | 214 | (7.0%) | 745 | (24.3%) | 66 | (2.2%) | 251 | (8.1%) | 96 | (3.1%) |
| Experience of warning for exceeding 5 mSv per quarter | | | | | | | | | | | | | | | | |
| No | 1660 | (9.8%) | 684 | (4.1%) | 159 | (1.0%) | 612 | (3.7%) | 1774 | (10.7%) | 129 | (0.8%) | 496 | (3.0%) | 280 | (1.7%) |
| Yes | 104 | (12.3%) | 35 | (4.2%) | 14 | (1.7%) | 25 | (3.1%) | 132 | (16.2%) | 14 | (1.7%) | 36 | (4.4%) | 26 | (3.2%) |
| Night shifts | | | | | | | | | | | | | | | | |
| None | 889 | (9.1%) | 396 | (4.1%) | 89 | (0.9%) | 331 | (3.5%) | 939 | (9.8%) | 77 | (0.8%) | 243 | (2.5%) | 156 | (1.6%) |
| 1–9 years | 682 | (9.0%) | 307 | (4.1%) | 61 | (0.8%) | 233 | (3.1%) | 725 | (9.7%) | 58 | (0.8%) | 223 | (3.0%) | 131 | (1.8%) |
| ≥10 years | 256 | (14.6%) | 61 | (3.6%) | 32 | (1.9%) | 101 | (6.1%) | 315 | (18.8%) | 19 | (1.1%) | 85 | (5.0%) | 32 | (1.9%) |
| While engaged in radiation work, white blood cell counts fell below the normal range | | | | | | | | | | | | | | | | |
| No | 1678 | (9.8%) | 674 | (4.0%) | 153 | (0.9%) | 577 | (3.5%) | 1816 | (10.9%) | 133 | (0.8%) | 501 | (3.0%) | 281 | (1.7%) |
| Yes | 50 | (11.0%) | 27 | (6.1%) | 14 | (3.2%) | 31 | (7.0%) | 60 | (13.5%) | 8 | (1.8%) | 17 | (3.8%) | 18 | (4.1%) |

*PR* prevalence rate, *BMI* body mass index
[a] Including hypertension, stroke, myocardial infarction, and angina
[b] Including pulmonary tuberculosis and asthma
[c] Including rheumatoid arthritis and osteoporosis
[d] Including benign thyroid tumor, thyroid nodule, thyroid goiter, thyroiditis, hyperthyroidism, and hypothyroidism

**Supplementary Table S2** Dose-response analysis of the prevalence of non-cancer diseases among radiation workers

| Diseases | | POR for dose categories (95% CI) (mSv) | | | | | | P-value of trend test | POR per 10 mSv (95% CI) |
|---|---|---|---|---|---|---|---|---|---|
| | | 0 (n = 7,901) | 0.10–0.99 (n = 3,155) | 1.00–4.99 (n = 3,040) | 5.00–19.99 (n = 3,164) | 20.00–49.99 (n = 1,934) | ≥50.00 (n = 1,414) | | |
| Diseases of the circulatory system[a] | | | | | | | | | |
| | Cases | 472 | 250 | 292 | 355 | 257 | 229 | | |
| | Crude | 1 | 1.35 (1.15–1.59) | 1.69 (1.45–1.96) | 2.01 (1.74–2.32) | 2.43 (2.07–2.85) | 3.10 (2.62–3.68) | <0.0001 | 1.08 (1.06–1.09) |
| | Adjusted[e] | 1 | 1.01 (0.84–1.22) | 0.99 (0.82–1.19) | 0.93 (0.76–1.13) | 1.07 (0.84–1.35) | 1.03 (0.78–1.35) | 0.8097 | 1.00 (0.98–1.02) |
| Diseases of the respiratory system[b] | | | | | | | | | |
| | Cases | 274 | 123 | 119 | 110 | 65 | 77 | | |
| | Crude | 1 | 1.12 (0.91–1.40) | 1.15 (0.92–1.43) | 1.02 (0.81–1.27) | 0.98 (0.75–1.29) | 1.65 (1.28–2.15) | 0.2756 | 1.03 (1.01–1.05) |
| | Adjusted | 1 | 1.06 (0.83–1.34) | 1.02 (0.79–1.31) | 0.84 (0.63–1.13) | 0.78 (0.54–1.12) | 1.11 (0.75–1.66) | 0.0602 | 1.01 (0.98–1.03) |
| Diseases of the musculoskeletal system[c] | | | | | | | | | |
| | Cases | 45 | 35 | 29 | 32 | 17 | 26 | | |
| | Crude | 1 | 1.95 (1.25–3.04) | 1.70 (1.07–2.72) | 1.81 (1.15–2.86) | 1.57 (0.90–2.76) | 3.38 (2.08–5.50) | 0.5285 | 1.07 (1.04–1.10) |
| | Adjusted | 1 | 1.77 (1.08–2.91) | 1.43 (0.83–2.46) | 1.19 (0.65–2.18) | 1.26 (0.60–2.66) | 2.32 (1.09–4.93) | 0.2957 | 1.03 (1.00–1.07) |
| Thyroid diseases[d] | | | | | | | | | |
| | Cases | 231 | 110 | 109 | 114 | 61 | 44 | | |
| | Crude | 1 | 1.20 (0.95–1.51) | 1.26 (1.00–1.59) | 1.26 (1.00–1.58) | 1.11 (0.83–1.48) | 1.12 (0.81–1.56) | 0.6411 | 1.02 (1.00–1.04) |
| | Adjusted | 1 | 0.98 (0.76–1.27) | 0.90 (0.69–1.19) | 0.76 (0.56–1.03) | 0.65 (0.44–0.97) | 0.48 (0.31–0.75) | 0.0245 | 0.96 (0.93–0.99) |
| Hyperlipidemia | | | | | | | | | |
| | Cases | 535 | 239 | 311 | 391 | 274 | 255 | | |
| | Crude | 1 | 1.13 (0.97–1.33) | 1.59 (1.38–1.85) | 1.99 (1.74–2.29) | 2.34 (2.00–2.73) | 3.23 (2.74–3.80) | <0.0001 | 1.09 (1.07–1.10) |
| | Adjusted | 1 | 0.89 (0.74–1.06) | 0.98 (0.82–1.17) | 0.94 (0.78–1.13) | 1.01 (0.80–1.26) | 1.08 (0.83–1.39) | 0.3657 | 1.00 (0.99–1.02) |
| Cataracts | | | | | | | | | |
| | Cases | 43 | 28 | 19 | 23 | 21 | 22 | | |
| | Crude | 1 | 1.63 | 1.16 | 1.36 | 2.04 | 2.98 | 0.3701 | 1.09 |

| | | | | | | | | | |
|---|---|---|---|---|---|---|---|---|---|
| | | | (1.01–2.62) | (0.68–2.00) | (0.82–2.26) | (1.21–3.45) | (1.78–5.00) | | (1.06–1.12) |
| | Adjusted | 1 | 1.13 | 0.61 | 0.54 | 0.74 | 0.75 | 0.2451 | 1.04 |
| | | | (0.65–1.95) | (0.32–1.16) | (0.28–1.05) | (0.36–1.56) | (0.33–1.69) | | (1.00–1.07) |
| Diabetes mellitus | | | | | | | | | |
| | Cases | 113 | 78 | 86 | 110 | 82 | 94 | | |
| | Crude | 1 | 1.74 | 2.03 | 2.53 | 3.10 | 5.05 | <0.0001 | 1.09 |
| | | | (1.30–2.33) | (1.53–2.68) | (1.94–3.30) | (2.32–4.14) | (3.82–6.68) | | (1.07–1.11) |
| | Adjusted | 1 | 1.30 | 1.15 | 1.05 | 1.26 | 1.30 | 0.7792 | 1.01 |
| | | | (0.94–1.81) | (0.82–1.61) | (0.74–1.50) | (0.84–1.90) | (0.83–2.05) | | (0.98–1.03) |
| Hepatitis | | | | | | | | | |
| | Cases | 87 | 39 | 46 | 63 | 50 | 37 | | |
| | Crude | 1 | 1.12 | 1.40 | 1.85 | 2.43 | 2.49 | <0.0001 | 1.06 |
| | | | (0.76–1.63) | (0.98–2.00) | (1.33–2.56) | (1.71–3.45) | (1.69–3.68) | | (1.03–1.08) |
| | Adjusted | 1 | 0.87 | 1.00 | 1.12 | 1.34 | 1.03 | 0.0837 | 0.98 |
| | | | (0.57–1.31) | (0.67–1.49) | (0.74–1.71) | (0.82–2.18) | (0.58–1.83) | | (0.95–1.02) |

*POR* prevalence odds ratio, *CI* confidence interval

[a] Including hypertension, stroke, myocardial infarction, and angina

[b] Including pulmonary tuberculosis and asthma

[c] Including rheumatoid arthritis and osteoporosis

[d] Including benign thyroid tumor, thyroid nodule, thyroid goiter, thyroiditis, hyperthyroidism, and hypothyroidism

[e] Prevalence odds ratios were adjusted for sex, age, occupation, duration of employment, smoking status, alcohol status, regular exercise, body mass index (BMI), and night-shift work
